# Prognostic implications of STK11 with different mutation status and its relationship with tumor-infiltrating immune cells in non-small cell lung cancer

**DOI:** 10.3389/fimmu.2024.1387896

**Published:** 2024-04-26

**Authors:** Jianqing Zheng, Yujie Deng, Bifen Huang, Xiaohui Chen

**Affiliations:** ^1^ Department of Radiation Oncology, The Second Affiliated Hospital of Fujian Medical University, Quanzhou, Fujian, China; ^2^ Department of Medical Oncology, The First Affiliated Hospital of Fujian Medical University, Fuzhou, Fujian, China; ^3^ Department of Obstetrics and Gynecology, Quanzhou Medical College People’s Hospital Affiliated, Quanzhou, Fujian, China; ^4^ Department of Thoracic Surgery, Clinical Oncology School of Fujian Medical University, Fujian Cancer Hospital, Fuzhou, China; ^5^ The Graduate School of Fujian Medical University, Fuzhou, Fujian, China; ^6^ Interdisciplinary Institute of Medical Engineering of Fuzhou University, Fuzhou, Fujian, China

**Keywords:** non-small cell lung cancer, STK11 gene, immune related genes, immunochemistry, prognostic analysis

## Abstract

**Background:**

Mutations in STK11 (STK11^Mut^) gene may present a negative impact on survival in Non-small Cell Lung Cancer (NSCLC) patients, however, its relationship with immune related genes remains unclear. This study is to unveil whether overexpressed- and mutated-STK11 impact survival in NSCLC and to explore whether immune related genes (IRGs) are involved in STK11 mutations.

**Methods:**

188 NSCLC patients with intact formalin-fixed paraffin-embedded (FFPE) tissue available for detecting STK11 protein expression were included in the analysis. After immunohistochemical detection of STK11 protein, patients were divided into high STK11 expression group (STK11^High^) and low STK11 expression group (STK11^Low^), and then Kaplan-Meier survival analysis and COX proportional hazards model were used to compare the overall survival (OS) and progression-free survival (PFS) of the two groups of patients. In addition, the mutation data from the TCGA database was used to categorize the NSCLC population, namely STK11 Mutated (STK11^Mut^) and wild-type (STK11^Wt^) subgroups. The difference in OS between STK11^Mut^ and STK11^Wt^ was compared. Finally, bioinformatics analysis was used to compare the differences in IRGs expression between STK11^Mut^ and STK11^Wt^ populations.

**Results:**

The median follow-up time was 51.0 months (range 3.0 - 120.0 months) for real-life cohort. At the end of follow-up, 64.36% (121/188) of patients experienced recurrence or metastasis. 64.89% (122/188) of patients ended up in cancer-related death. High expression of STK11 was a significant protective factor for NSCLC patients, both in terms of PFS [HR=0.42, 95% CI= (0.29-0.61), *P*<0.001] and OS [HR=0.36, 95% CI= (0.25, 0.53), *P*<0.001], which was consistent with the finding in TCGA cohorts [HR=0.76, 95%CI= (0.65, 0.88), *P*<0.001 HR=0.76, 95%CI= (0.65, 0.88), *P*<0.001]. In TCGA cohort, STK11 mutation was a significant risk factor for NSCLC in both lung squamous cell carcinoma (LUSC) and lung adenocarcinoma (LUAD) histology in terms of OS [HR=6.81, 95%CI= (2.16, 21.53), *P*<0.001; HR=1.50, 95%CI= (1.00, 2.26), *P*=0.051, respectively]. Furthermore, 7 IRGs, namely CALCA, BMP6, S100P, THPO, CGA, PCSK1 and MUC5AC, were found significantly overexpressed in STK11-mutated NSCLC in both LUSC and LUAD histology.

**Conclusions:**

Low STK11 expression at protein level and presence of STK11 mutation were associated with poor prognosis in NSCLC, and mutated STK11 might probably alter the expression IRGs profiling.

## 1 Introduction

Lung cancer (LC) is one of the most common malignant tumors that pose the greatest threat to human health and life worldwide, with a high morbidity and mortality (1). The incidence rate of lung cancer in men and women is 12% and 13% respectively, and the mortality rate accounts for 22%, far exceeding other types of cancer and ranking first in cancer deaths (2). Among all types of LC, non-small cell lung cancer (NSCLC) accounts for 85%, and most patients are diagnosed at an advanced stage (3), which hence turned out a poor prognosis (4). In recent years, significant progress has been made in the treatment of NSCLC, with a significant reduction in patient mortality (5). In addition to targeted treatment for patients with sensitive oncogenic driver gene, the use of immune checkpoint inhibitors (ICIs) in NSCLC has greatly improved patient prognosis (6) and was thus approved by FDA as the state-of-the-art regimen either in the posterior-line or perioperative settings.

The serine threonine kinase 11 (STK11) gene encoded liver kinase B1 (LKB1), a highly conserved serine/threonine kinase involved in many energy-related cellular processes (7). Somatic mutations in STK11 often occur in NSCLC, however, its roles in immune- and targeted therapy remains unclear (8). The STK11 mutation defines a special subtype of lung adenocarcinoma (LUAD) patients, and emerging evidences suggested that STK11 alterations may be prognostic and/or predictive of therapeutic response, particularly in immune- and targeted therapy, and some studies demonstrated that loss of function caused by STK11 mutation was highly correlated with poor outcomes of NSCLC (9–11). STK11 mainly encodes serine threonine kinase, which regulates cell metabolism, energy homeostasis, cell growth, etc. through AMPK signaling pathway and 12 AMPK related kinases (12). The mutation rate of STK11 in lung adenocarcinoma was 16.7%, and the co-mutation rate with KRAS was 25.4% (12, 13). The inactivation of STK11 gene or its protein product LKB1 is related to the cold tumor immune environment, which is accompanied by the decrease of infiltrating cytotoxic CD8+ T lymphocytes in both human tumors and genetically engineered mouse models (14). Karatrasoglou et al. have found that there is an interaction between gene mutations and abnormal activation of pd-1/pd-l1 signaling in LC (15). For LC patients with positive driver genes (EGFR mutations, ALK fusion, ROS1 fusion, etc.), immune checkpoint inhibitors are less effective (15). Increasing evidence shows that NSCLC exhibits significant clinical heterogeneity, and currently, a single oncogenic driver has not been fully explained (16). Therefore, future research and ongoing clinical trials will help us better understand the role of STK11 in cancer development and develop more effective treatment strategies.

In the present study, we first conducted a retrospective study to evaluate the prognostic value of STK11 in a FFPE specimen cohort, second explored the prognostic differences between wild-type and mutated STK11 NSCLC patients through TCGA databases, then third compared the differentially expressed genes related to immunotherapy between the two subtypes of patients via bioinformatic analyses. The future development direction of this field was also discussed, with a view to provide evidence that is more in line with STK11 in targeted therapy and immunotherapy for NSCLC.

## 2 Materials and methods

### 2.1 Study protocol

**Figure 1:**
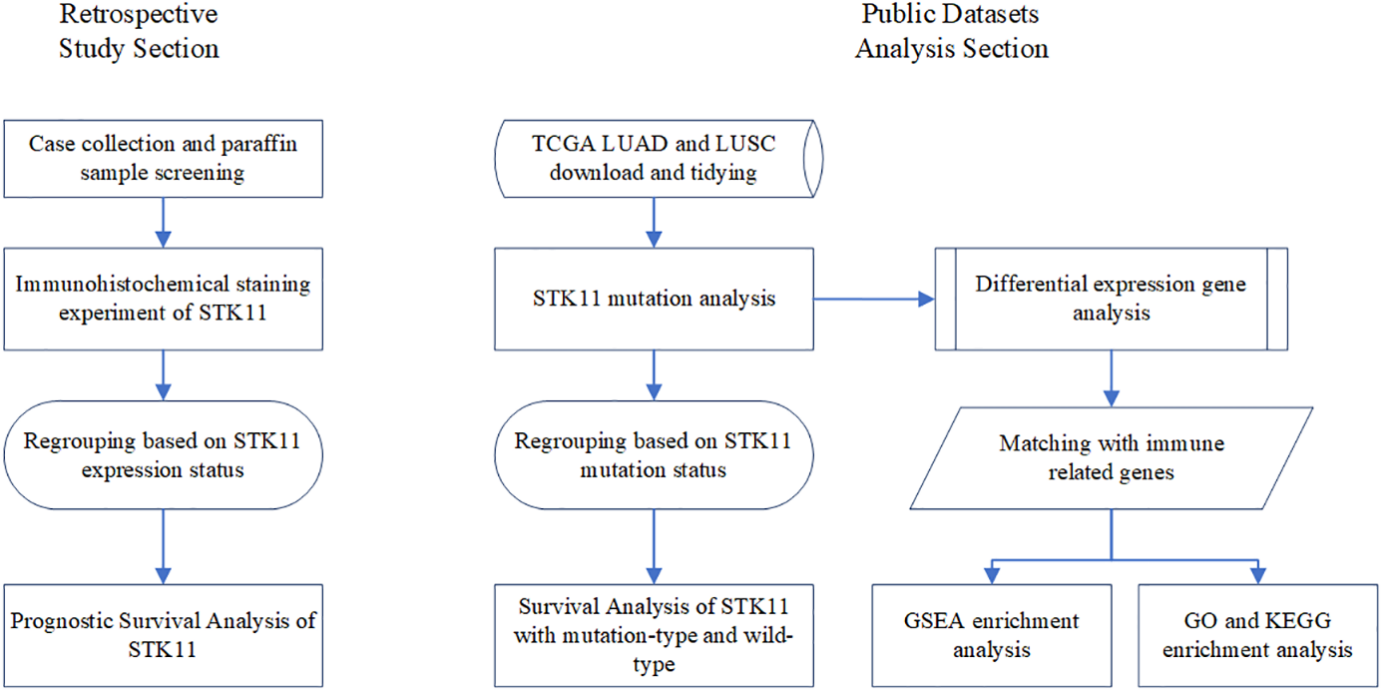
Flow chart of study design.

The current study consists of two parts, one of which explores the prognostic value of different levels of STK11 expression based on immunohistochemistry and survival information of 188 lung cancer patients from Fujian Medical University Cancer Hospital, and the other explores the prognosis of different STK11 change states through bioinformatics analysis. Recruited into this study were a total of 188 patients admitted to Fujian Medical University Cancer Hospital between January 2010 and July 2011. All paraffin tissue originates from donations from surgical patients, and written informed consent was provided from each donor. The survival information for each participant comes from case records, telephone follow-up, and official death records. The study was approved by the ethics committee of Fujian Medical University Cancer Hospital (SQ2021-101-01). The flowchart of this study is shown in **Figure 1**.

Included cases in this study were patients who (1) had pathologically confirmed NSCLC and had undergone radical resection; (2) were in TNM stages I, II, III according to the 8^th^ American Joint Committee on Cancer; (3) were aged between 18 and 80 years old; (4) had Karnofsky’s performance score ≥70; (4) had given informed consent and detailed follow-up records. The exclusion criteria included stage IV; any preoperative treatment, including radiotherapy, chemotherapy, or immunotherapy, etc; and double or multiple cancers. The clinicopathological data of included patients were collected, and the immunohistochemical experiment of STK11 was performed. All the patients were routinely followed up and survival information was recorded to establish a follow-up database. Progression-free survival (PFS) is defined as the time span from the date of surgery to the first occurrence of recurrence, metastasis, or death. Overall survival (OS) was defined as the period from the date of surgery to the date of death or final follow-up. The follow-up was censored on 30 December 2021.

### 2.2 Immunohistochemistry assay of STK11

The 4-µm thick FFPE NSCLC tissue sections were re-cut from samples stored in pathology department and mounted on Silane-coated slides. Immunohistochemical staining was conducted in an automatic staining machine (Rapid High throughput Intelligent Immunohistochemical Staining Machine CNT360, Sanofit). Antigen recovery was carried out in a hot 10mm sodium citrate buffer at pH 6.0, gradually increasing from 50°C to 100°C in a microwave oven for 40 minutes. A rabbit polyclonal antibody to STK11 (EPR19379, ab199970, Abcam) was used for immunostaining. The STK11 antibody was used at a dilution of 1:500, incubated for 45 minutes, and then the Vector Universal Elite ABC immunohistochemistry kit (secondary antibody dilution of 1:100) was used, with DAB as the chromogenic agent. Normal para-cancerous tissue was used as a positive normal control for the marker. Evaluation of STK11 expression Sections were examined and evaluated microscopically by the two independent researchers. The intensity of immunohistochemical expression of STK11 was graded on a scale of 0–3 as follows: 0 = no staining; 1 = week intensity, 2 = moderate intensity, and 3 = strong intensity. Positive immunohistochemical staining cells were counted at 5 high-power fields on each slice (×200). The number of positive cells was graded on a scale of 0 points for less than 5%, 1 point for 5% to 25%, 2 points for 26% to 50%, 3 points for 51% to 75% and 4 points for 76% to 100%. The multiplication of the intensity score and positive score results in a positive rating: 0 points are negative (-), 1-4 points are weakly positive (+), 5-8 points are positive (++), and 9-12 points are strongly positive (+++). All lung cancer patients were regrouped based on a positive rating score of 5, where <5 points indicated the low-expression group (LE group, STK11^Low^ group) and ≥5 points indicated the high-expression group (HE group, STK11^High^ group).

### 2.3 Analysis based on public datasets

The transcriptome RNA-Seq raw counts of NSCLC patients were downloaded from TCGA-LUAD and TCGA-LUSC [https://portal.gdc.cancer.gov (accessed on 23 October 2023)]. Corresponding genes expression information and clinical information was extracted and merged in R 4.3.1. The downloaded samples were divided into STK11 mutation-type and wild-type via “TCGAmutations” and “maftools” R package. The “limma” R package was used to screen differentially expressed genes (DEGs) between STK11 mutation-type and STK11 wild-type, with |Fold Change| ≥1 and p-value < 0.05 as the cutoff value. A immune related genes list was downloaded from previously published available literature (17). The gene enrichment analysis method based on gene set enrichment analysis (GSEA) was used to analyze the potential functions of genes. The Kaplan Meier plot database was used to explore the prognostic value of the STK11 gene (18).

### 2.4 Statistical analysis

The associations between STK11 and clinicopathological parameters were illustrated using independent-sample t test, Chi square test or Fisher’s exact test. For survival variables (such as OS and DFS), HR and its corresponding 95% CIs were applied as the effect size. The Kaplan-Meier curves were plotted by log-rank tests, and prognostic value of STK11 was analyzed using the Cox proportional risk model via “survminer” and “survival” R package. A random forest model was conducted via “randomForestSRC” R package. ssGSEA was used for immune infiltration analysis. p-values less than 0.05 were considered statistically significant. The above analyses were performed on R (version 4.3.1).

## 3 Results

### 3.1 Clinicopathological characteristics of patients

**Figure 2:**
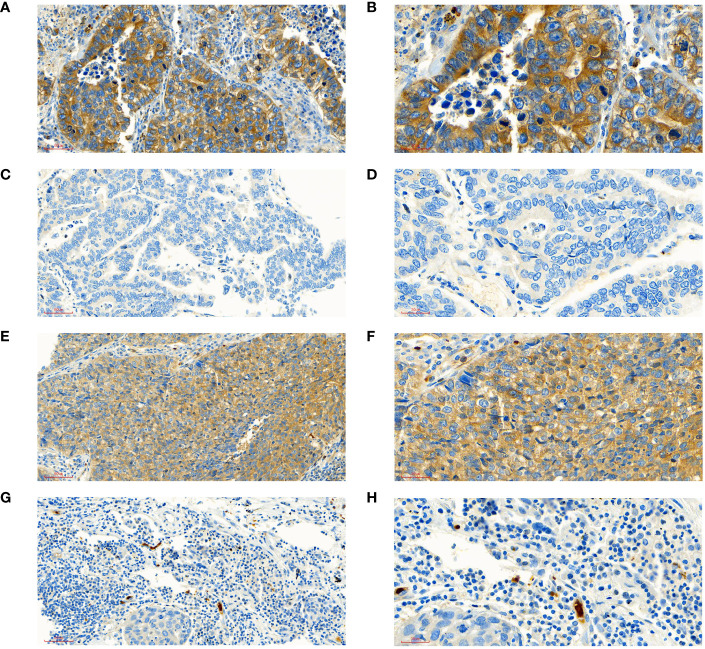
Interpretation of STK11 in immunohistochemical staining. **(A)** ADE patient with high expression of STK11 (intensity score=3, positive score=4, positive rating=12, under ×20 field). **(B)** ADE patient with high expression of STK11 (intensity score=3, positive score=4, positive rating=12, under ×40 field, come from the same patient with A). **(C)** ADE patient with negative expression of STK11 (intensity score=0, positive score=0, positive rating=0, under ×20 field). **(D)** ADE patient with negative expression of STK11 (intensity score=0, positive score=0, positive rating=0, under ×40 field, come from the same patient with C). **(E)** SCC patient with high expression of STK11 (intensity score=3, positive score=4, positive rating=12, under ×20 field). **(F)** SCC patient with high expression of STK11 (intensity score=3, positive score=4, positive rating=12, under ×40 field, come from the same patient with E). **(G)** SCC patient with negative expression of STK11 (intensity score=0, positive score=0, positive rating=0, under ×20 field). **(H)** SCC patient with negative expression of STK11 (intensity score=0, positive score=0, positive rating=0, under ×40 field, come from the same patient with G) (ADE, Lung adenocarcinoma; SCC, Lung squamous cell carcinoma).

A total of 188 patients with NSCLC were enrolled, including 104 (55.32%) patients in low-expression group and 84 (44.68%) patients in high-expression group. Of them, 50 (26.60%) patients were at stage IA/IB, 57(30.32%) at stage IIA/IIB and 81(43.09%) at stage IIIA/IIIB; 70(37.23%) patients were aged 60 years or older; 128 (68.09%) patients were male. 65 (34.57%) had received video assisted thoracoscopic surgery (VATS). 37 (19.68%) patients had completed adjuvant radiotherapy and 98 (52.13%) patients had completed adjuvant chemotherapy. The intensity score of STK11 were (0.75 ± 0.66) points and (2.48 ± 0.50) points in LE group and HE group, respectively, with significant differences (t= 10.072, *P*<0.001). The score of positive percentage of STK11 were (2.10 ± 1.81) points and (3.92 ± 0.32) points in LE group and HE group, respectively, with significant differences (t= 20.269, *P*<0.001). The score of positive rating of STK11 were (2.32 ± 1.88) points and (9.69 ± 2.11) points in LE group and HE group, respectively, with significant differences (t=24.984, *P*<0.001). The immunohistochemical staining results of STK11 in 2 cases of LUAD and 2 cases of LUSC are shown in **Figure 2**, with 1 case of LUAD and 1 case of LUSC showing strong expression, and the other 2 cases showing negative expression. The comparative information between the two groups of patients was displayed in **Table 1**. The panoramic data of the patients included in the study were presented in **Supplementary Table S1**.

**Table 1:** Clinicopathological characteristics in NSCLC.

Parameters	Levels	Total	Low-expression group(n=104)	High-expression group(n=84)	Statistic	*P*
STK11 Status	Negative	39(20.74)	39(37.50)	0(0.00)	188.000	<0.001
	Weakly positive	65(34.57)	65(62.50)	0(0.00)		
	Positive	45(23.94)	0(0.00)	45(53.57)		
	Strong positive	39(20.74)	0(0.00)	39(46.43)		
TNM Stage	Stage IA/IB	50(26.60)	29(27.88)	21(25.00)	0.324	0.850
	Stage IIA/IIB	57(30.32)	32(30.77)	25(29.76)		
	Stage IIIA/IIIB	81(43.09)	43(41.35)	38(45.24)		
Age		58.13 ± 9.10	57.28 ± 9.27	59.19 ± 8.82	1.437	0.153
Age group	Younger	118(62.77)	71(68.27)	47(55.95)	3.016	0.082
	Older	70(37.23)	33(31.73)	37(44.05)		
Gender	Female	60(31.91)	28(26.92)	32(38.10)	2.669	0.102
	Male	128(68.09)	76(73.08)	52(61.90)		
VATS	No	123(65.43)	69(66.35)	54(64.29)	0.087	0.768
	Yes	65(34.57)	35(33.65)	30(35.71)		
Smoking Status	Never-smoker	95(50.53)	53(50.96)	42(50.00)	0.017	0.896
	Smoker	93(49.47)	51(49.04)	42(50.00)		
Postoperative Radiotherapy	No	151(80.32)	86(82.69)	65(77.38)	0.829	0.362
	Yes	37(19.68)	18(17.31)	19(22.62)		
Postoperative Chemotherapy	No	90(47.87)	52(50.00)	38(45.24)	0.422	0.516
	Yes	98(52.13)	52(50.00)	46(54.76)		
Complications	No	179(95.21)	98(94.23)	81(96.43)	0.128	0.720
	Pneumonia	9(4.79)	6(5.77)	3(3.57)		
Pathological Type	Squamous cell carcinoma	75(39.89)	38(36.54)	37(44.05)	1.093	0.296
	Adenocarcinoma	113(60.11)	66(63.46)	47(55.95)		
Comorbidities	No	161(85.64)	88(84.62)	73(86.90)	4.170	0.244
	Hypertension	19(10.11)	9(8.65)	10(11.90)		
	Diabetes	5(2.66)	4(3.85)	1(1.19)		
	Others	3(1.60)	3(2.88)	0(0.00)		
STK11- intensity		1.52 ± 1.05	0.75 ± 0.66	2.48 ± 0.50	20.269	<0.001
STK11-Positive percentage		2.91 ± 1.63	2.10 ± 1.81	3.92 ± 0.32	10.072	<0.001
STK11-score		5.61 ± 4.18	2.32 ± 1.88	9.69 ± 2.11	24.984	<0.001
SmokingIndex		410.80 ± 762.67	428.37 ± 944.18	389.05 ± 450.92	0.375	0.708
BMI		23.33 ± 3.10	22.94 ± 3.03	23.81 ± 3.14	1.911	0.058

VATS, video assisted thoracoscopic surgery; BMI, body mass index.

### 3.2 Prognostic analysis of STK11 between LE group and HE group

**Figure 3:**
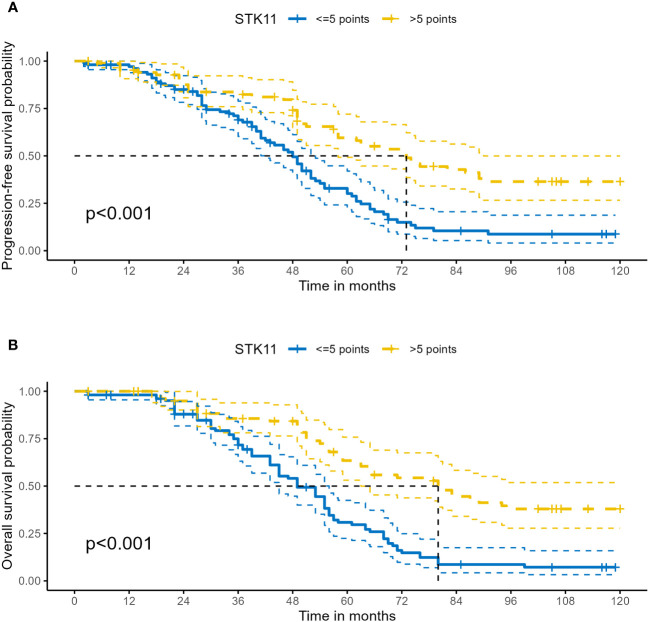
Kaplan–Meier survival analysis of STK11 expression on survival in human LC. **(A)** PFS of STK11 expression on LC. **(B)** OS of STK11 expression on LC (LC, lung cancer; PFS, progression-free survival; OS, overall survival).

**Figure 4:**
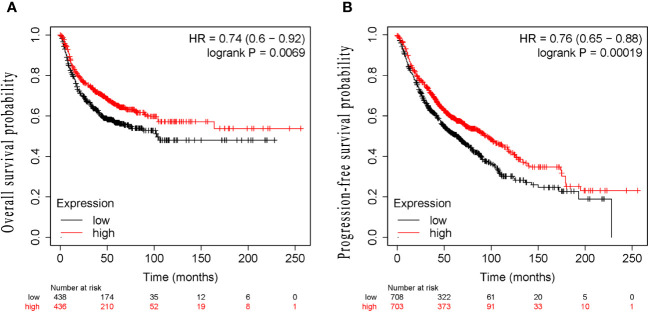
Kaplan–Meier survival analysis of STK11 expression on survival in human LC from Kaplan-Meier plotter. **(A)** PFS of STK11 expression on LC. **(B)** OS of STK11 expression on LC (LC, lung cancer; PFS, progression-free survival; OS, overall survival).

**Figure 5:**
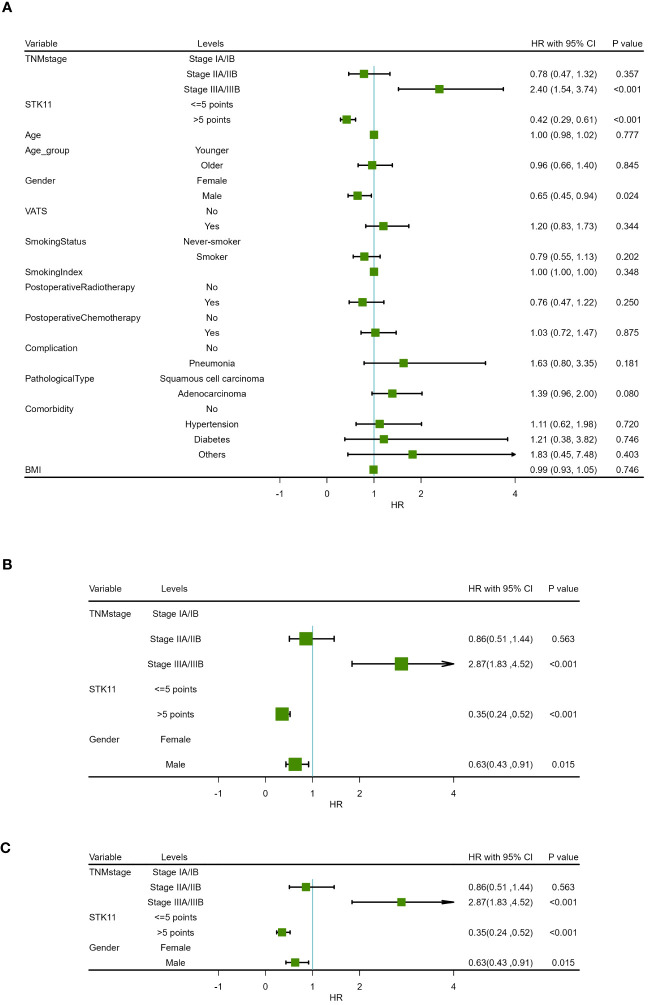
Forest map of progression-free survival in COX proportional-hazards model in human lung cancer. **(A)** Results of univariate prognostic analysis. **(B)** Results of multivariate prognostic analysis with all significant variables. **(C)** Results of multivariate prognostic analysis via stepwise regression.

**Figure 6:**
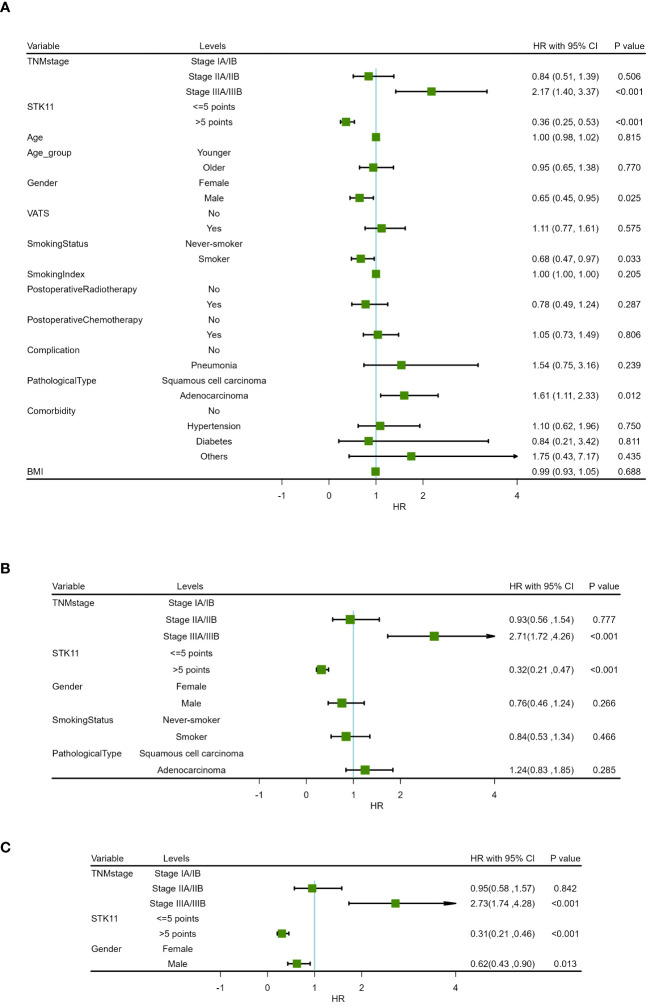
Forest map of overall survival in COX proportional-hazards model in human lung cancer. **(A)** Results of univariate prognostic analysis. **(B)** Results of multivariate prognostic analysis with all significant variables. **(C)** Results of multivariate prognostic analysis via stepwise regression.

The median follow-up time was 51.0 months (range 3.0–120.0months) for all patients. At the end of follow-up, 64.36% (121/188) of patients experienced recurrence or metastasis. 64.89% (122/188) of patients suffered from death. The clinical characteristics of cancer related deaths are summarized in **Table 2**. Cancer related death was occurred in 113(60.11%) patients, of whom 73(70.19%) was occurred in LE group and 40(47.62%) in HE group. Kaplan-Meier survival analysis showed that progression-free survival proportion at 3-years and 5-years were 68.94% (60.27%-78.86%) vs 83.69% (75.95%-92.23%) and 30.13% (21.63%-41.97%) vs 59.54% (49.24%-72.00%), respectively, for LE group and HE group. Similarly, overall survival proportion at 3-years and 5-years were 71.65% (63.12%-81.34%) vs 85.61% (78.08%-93.86%) and 30.85% (22.41%-42.47%) vs 63.44% (53.10%-75.80%), respectively, for LE group and HE group. Kaplan-Meier curves of PFS and OS were shown in **Figures 3A, B**. Additionally, data from Kaplan-Meier plotter showed that high expression of STK11 was a significant protective factor for NSCLC patients, both in terms of PFS (HR=0.74, 95%CI= (0.60-0.92), *P*=0.007) and OS (HR=0.76, 95%CI= (0.65, 0.88), *P*<0.001) (18), as shown in **Figures 4A, B**. Univariate Cox proportional-hazards model showed that high expression of STK11 was a significant protective factor for LC patients, both in terms of PFS (HR=0.42, 95%CI= (0.29-0.61), *P*<0.001) and OS (HR=0.36, 95%CI= (0.25, 0.53), *P*<0.001), as shown in **Figures 5A**, **6A**.

**Table 2:** The clinical characteristics of cancer related deaths.

Parameters	Levels	Total	Low-expression group(n=104)	High-expression group(n=84)	Statistic	*P*
Overall survival	Alive	66(35.11)	24(23.08)	42(50.00)	14.785	<0.001
	Dead	122(64.89)	80(76.92)	42(50.00)		
Progression-free survival	Progression-free	67(35.64)	28(26.92)	39(46.43)	7.708	0.005
	Progression	121(64.36)	76(73.08)	45(53.57)		
Death analysis	Cancer related death	113(60.11)	73(70.19)	40(47.62)	15.956	0.001
	Non-cancer death	9(4.79)	7(6.73)	2(2.38)		
	Survival with cancer	5(2.66)	1(0.96)	4(4.76)		
	Survival without cancer	61(32.45)	23(22.12)	38(45.24)		

In addition, univariate Cox proportional-hazards model showed that TNM stage and gender had a significant impact on PFS (*P*<0.05), and TNM stage, gender smoking status and pathological type have a significant impact on OS(*P*<0.05). Therefore, two multivariate COX models were developed to detect the prognostic value of STK11. The first multivariate model included all of the significant univariates in **Tables 3**, **4**, while the second model was analyzed using stepwise regression based on the first model. The results of the first model are shown in **Supplementary Tables S2**, **S3**; **Figures 5B**, **6B**. The results of the second model are shown in **Tables 5**, **6**. In the stepwise regression multivariate model, STK11 has a significant impact on both PFS (HR=0.31, 95%CI= (0.24,0.52), *P*<0.001) and OS (HR=0.31, 95%CI= (0.21,0.46), as shown in **Figures 5C**, **6C**.

**Table 3:** Univariate analysis results of progression-free survival in COX proportional hazards model.

Characteristics	Levels	Beta	SE	HR (95% CI for HR)	Statistics(Z value)	*P*
TNM stage	Stage IA/IB					
	Stage IIA/IIB	-0.24	0.27	0.78 (0.47-1.32)	-0.920	0.357
	Stage IIIA/IIIB	0.87	0.23	2.40 (1.54-3.74)	3.855	<0.001
STK11	<=5 points					
	>5 points	-0.87	0.19	0.42 (0.29-0.61)	-4.509	<0.001
Age		0.00	0.01	1.00 (0.98-1.02)	0.283	0.777
Age group	Younger					
	Older	-0.04	0.19	0.96 (0.66-1.40)	-0.195	0.845
Gender	Female					
	Male	-0.43	0.19	0.65 (0.45-0.94)	-2.261	0.024
VATS	No					
	Yes	0.18	0.19	1.20 (0.83-1.73)	0.947	0.344
Smoking Status	Never-smoker					
	Smoker	-0.23	0.18	0.79 (0.55-1.13)	-1.275	0.202
Smoking Index		-0.00	0.00	1.00 (1.00-1.00)	-0.939	0.348
Postoperative Radiotherapy	No					
	Yes	-0.28	0.24	0.76 (0.47-1.22)	-1.149	0.250
Postoperative Chemotherapy	No					
	Yes	0.03	0.18	1.03 (0.72-1.47)	0.157	0.875
Complication	No					
	Pneumonia	0.49	0.37	1.63 (0.80-3.35)	1.339	0.181
Pathological Type	Squamous cell carcinoma					
	Adenocarcinoma	0.33	0.19	1.39 (0.96-2.00)	1.748	0.080
Comorbidity	No					
	Hypertension	0.11	0.30	1.11 (0.62-1.98)	0.358	0.720
	Diabetes	0.19	0.59	1.21 (0.38-3.82)	0.324	0.746
	Others	0.60	0.72	1.83 (0.45-7.48)	0.837	0.403
BMI		-0.01	0.03	0.99 (0.93-1.05)	-0.324	0.746

**Table 4:** Univariate analysis results of overall survival in COX proportional hazards model.

Characteristics	Levels	Beta	SE	HR (95% CI for HR)	Statistics(Z value)	P
TNM stage	Stage IA/IB					
	Stage IIA/IIB	-0.17	0.25	0.84 (0.51-1.39)	-0.665	0.506
	Stage IIIA/IIIB	0.78	0.22	2.17 (1.40-3.37)	3.457	<0.001
STK11	<=5 points					
	>5 points	-1.01	0.20	0.36 (0.25-0.53)	-5.168	<0.001
Age		0.00	0.01	1.00 (0.98-1.02)	0.234	0.815
Age group	Younger					
	Older	-0.06	0.19	0.95 (0.65-1.38)	-0.292	0.770
Gender	Female					
	Male	-0.43	0.19	0.65 (0.45-0.95)	-2.239	0.025
VATS	No					
	Yes	0.11	0.19	1.11 (0.77-1.61)	0.561	0.575
Smoking Status	Never-smoker					
	Smoker	-0.39	0.18	0.68 (0.47-0.97)	-2.137	0.033
Smoking Index		-0.00	0.00	1.00 (1.00-1.00)	-1.269	0.205
Postoperative Radiotherapy	No					
	Yes	-0.25	0.24	0.78 (0.49-1.24)	-1.065	0.287
Postoperative Chemotherapy	No					
	Yes	0.04	0.18	1.05 (0.73-1.49)	0.246	0.806
Complication	No					
	Pneumonia	0.43	0.37	1.54 (0.75-3.16)	1.178	0.239
Pathological Type	Squamous cell carcinoma					
	Adenocarcinoma	0.47	0.19	1.61 (1.11-2.33)	2.506	0.012
Comorbidity	No					
	Hypertension	0.09	0.29	1.10 (0.62-1.96)	0.319	0.750
	Diabetes	-0.17	0.71	0.84 (0.21-3.42)	-0.239	0.811
	Others	0.56	0.72	1.75 (0.43-7.17)	0.781	0.435
BMI		-0.01	0.03	0.99 (0.93-1.05)	-0.402	0.688

**Table 5:** Multivariate analysis results of progression-free survival in COX proportional risk model (Stepwise regression).

Characteristics	Levels	Beta	SE	HR (95% CI for HR)	Statistics(Z value)	*P*
TNMstage	Stage IA/IB					
	Stage IIA/IIB	-0.15	0.27	0.86(0.51,1.44)	0.579	0.563
	Stage IIIA/IIIB	1.06	0.23	2.87(1.83,4.52)	4.558	<0.001
STK11	<=5 points					
	>5 points	-1.04	0.20	0.35(0.24,0.52)	5.256	<0.001
Gender	Female					
	Male	-0.46	0.19	0.63(0.43,0.91)	2.442	0.015

**Table 6:** Multivariate analysis results of overall survival in COX proportional risk model (Stepwise regression).

Characteristics	Levels	Beta	SE	HR (95% CI for HR)	Statistics(Z value)	P
TNMstage	Stage IA/IB					
	Stage IIA/IIB	-0.05	0.26	0.95(0.58,1.57)	0.199	0.842
	Stage IIIA/IIIB	1.00	0.23	2.73(1.74,4.28)	4.363	<0.001
STK11	<=5 points					
	>5 points	-1.18	0.20	0.31(0.21,0.46)	5.863	<0.001
Gender	Female					
	Male	-0.47	0.19	0.62(0.43,0.90)	2.489	0.013

In order to get the importance of different prognostic variables and improve the ability of clinical applications, a machine learning model via random forest model was developed. With the 5 variables from univariate Cox proportional-hazards model, a random forest decision tree was shown in **Supplementary Figure S1**. In 188 cases of patients, 122 of cases of deaths occurred and 100 trees were built in random forest model. The requested performance error was 0.329 for our random forest model. The requested performance error and variable importance were shown in **Supplementary Figure S2**. Estimates of survival for random forest model were shown in **Supplementary Figure S3**. Brier score versus time is shown in **Supplementary Figure S4**. The variable importance was shown in **Supplementary Figure S5**. As shown in **Supplementary Figure S5**, STK11 and TNM stage were the most important predictors of survival.

### 3.3 Analysis of mutations of STK11 gene in lung cancer patients

**Figure 7:**
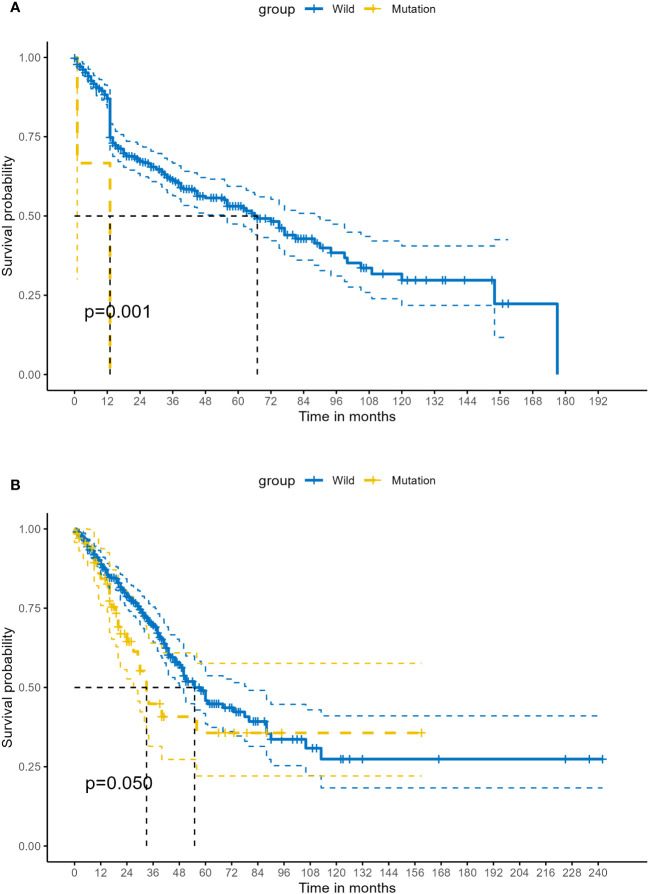
Kaplan–Meier survival analysis of different status of STK11 on survival in human LC. **(A)** OS of wild-type and mutation-type STK11 on LUSC. **(B)** OS of wild-type and mutation-type STK11 on LUAD. (LUSC, Lung squamous cell carcinoma; LUAD, Lung adenocarcinoma).

After integrating the TCGA mutation data and expression data of LUSC, 482 samples were successfully matched, of whom, 1.04% (5/482) patients were found to have STK11 mutations. And for LUAD, 517 samples were successfully matched, of whom, 16.70% (74/443) patients were found to have STK11 mutations. There is a significant difference in STK11 gene mutations between LUSC and LUAD (=59.249, *P*<0.001). A total of 88 mutations were found in 74 patients. In terms of mutation types, no synonymous mutations were found in the TCGA dataset. The most common types of mutations in STK11 include missense mutation (31.82%, 28/88), nonsense mutation (31.82%, 28/88), frameshift deletion mutation (17.05%, 15/88), splice site (13.64%, 12/88) and frameshift insertion mutation (5.68%, 5/88). The above mutations have moderate and strong impact on the expression product of STK11. Kaplan-Meier survival analysis showed that overall survival proportion at 1-years were 86.96% (83.81%-90.24%) vs 66.67% (29.95%-100.00%), respectively, for STK11 wild-type LUSC patients and STK11 mutation-type LUSC patients. Kaplan-Meier curves of OS were shown in **Figure 7A**. Univariate Cox proportional-hazards model showed that mutation-type STK11 was a significant risk factor for LUSC patients in terms of OS (HR=6.81, 95%CI= (2.16, 21.53), *P*<0.001). Kaplan-Meier survival analysis showed that overall survival proportion at 3-years and 5-years were 69.58% (64.21%-75.39%) vs 44.85% (31.44%-63.98%) and 45.85% (38.49%-54.63%) vs 35.67% (22.09%-57.61%), respectively, for STK11 wild-type LUAD patients and STK11 mutation-type LUAD patients. Kaplan-Meier curves of OS were shown in **Figure 7B**. Univariate Cox proportional-hazards model showed that mutation-type STK11 was a significant risk factor for LUAD patients in terms of OS (HR=1.50, 95%CI= (1.00,2.26), *P*=0.051).

### 3.4 Analysis of differentially expressed genes in different STK11 status in lung cancer patients

**Figure 8:**
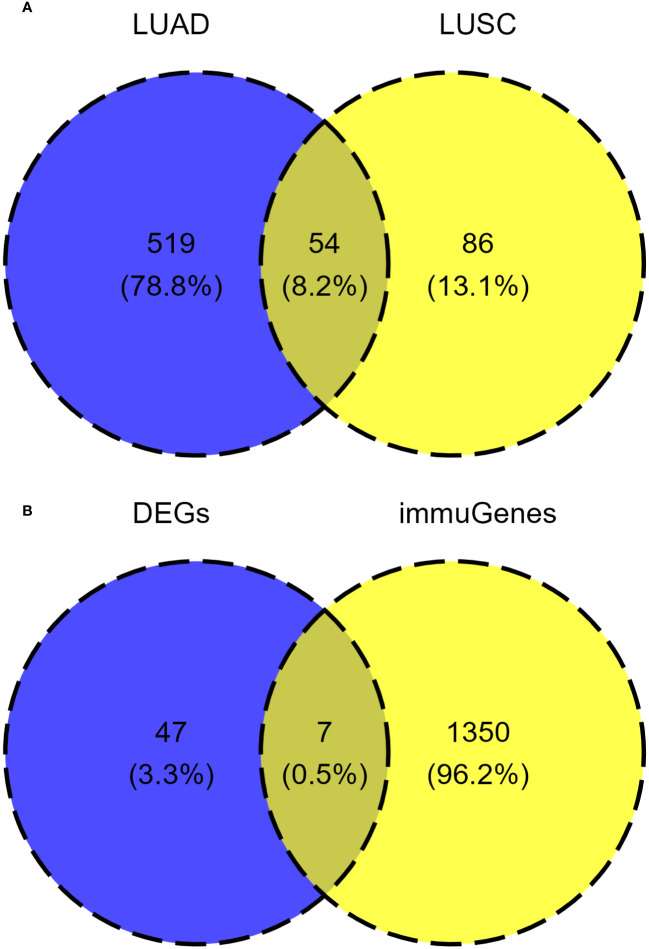
Analysis of common differentially expressed genes in different STK11 status between LUSC cohort and LUAD cohort. **(A)** Venn diagram of differentially expressed genes in the LUSC cohort and LUAD cohort. **(B)** Venn diagram of common differential expression of immune related genes. (LUSC, Lung squamous cell carcinoma; LUAD, Lung adenocarcinoma; DEGs, differentially expressed genes; immuGenes, immune related genes).

**Figure 9:**
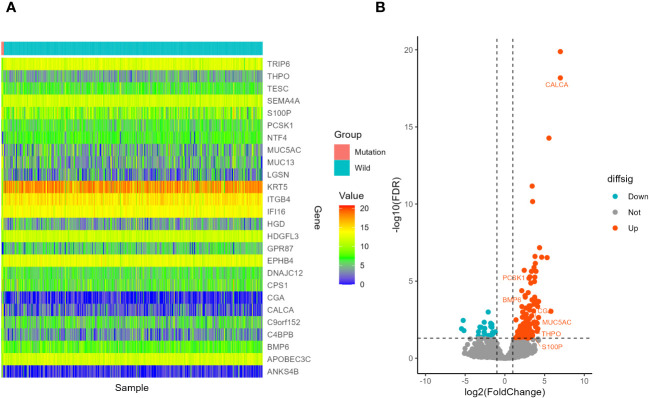
Screening of differentially expressed genes in different STK11 status in LUSC cohort. **(A)** Heatmap. The figure shows 10 genes with the most significant upregulation, 10 genes with the most significant downregulation, and 7 differentially expressed immune related genes, totaling 26 genes. **(B)** volcano plot. 7 differentially expressed immune related genes were selected to labeled.

**Figure 10:**
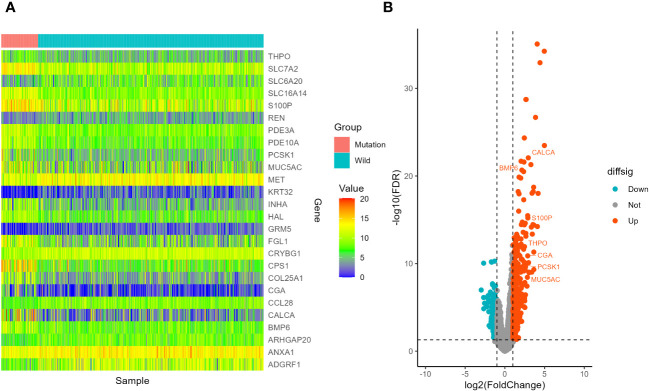
Screening of differentially expressed genes in different STK11 status in LUAD cohort. **(A)** Heatmap. The figure shows 10 genes with the most significant upregulation, 10 genes with the most significant downregulation, and 7 differentially expressed immune related genes, totaling 26 genes. **(B)** volcano plot. 7 differentially expressed immune related genes were selected to labeled.

In the LUSC cohort, a total of 29 genes were downregulated and 111 genes were upregulated. In the LUAD cohort, a total of 220 genes were downregulated and 353 genes were upregulated. Among all differentially expressed genes, a total of 54 genes co-existed in the LUSC cohort and LUAD cohort. The differential expression analysis (DEA) results of genes are shown in the **Supplementary Tables S4-S6**. Furthermore, matching with the immune gene list reported in previous literature, we identified 7 significantly differentially expressed immune related genes, which are listed in **Supplementary Table S7**. The 7 IRGs were CALCA, BMP6, S100P, THPO, CGA, PCSK1 and MUC5AC. The results of differential expression analysis are shown in **Figures 8**–**10**. All of the 7 IRGs were significantly overexpressed in STK11-mutated lung cancer, both in the LUSC cohort and LUAD cohort.

### 3.5 Enrichment analysis of differentially expressed genes in different STK11 status in lung cancer patients

**Figure 11:**
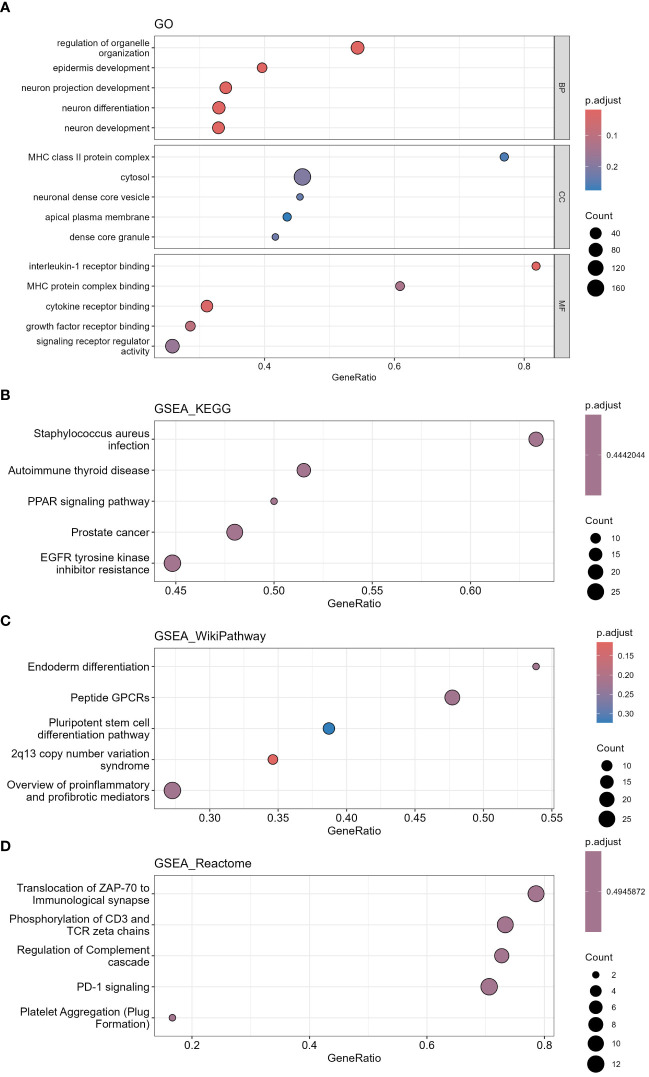
Functional annotation and pathway enrichment analysis of differentially expressed mRNAs in LUSC cohort via GSEA. **(A)** GO functional annotation, **(B)** KEGG pathway enrichment, **(C)** Wiki pathway enrichment, **(D)** Reactome pathway enrichment. (GO, Gene Ontology; KEGG, Kyoto Encyclopedia of Genes and Genomes; GSEA, Gene Set Enrichment Analysis).

Here, 1205 mRNAs related to immune genes in LUSC cohort were analyzed for GO functional annotation and KEGG pathway enrichment using the R package “clusterProfiler” via GSEA, and the results of the top 5 pathways for each enrichment analysis are shown in **Figure 11** and **Supplementary Table S8**. The main five enrichment pathways for GO biological processes (BP) were nervous system development, epidermis development, regulation of nervous system development, regulation of organelle organization and neuron projection development. The main five enrichment pathways of GO molecular function (MF) were interleukin-1 receptor binding, cytokine receptor binding, growth factor receptor binding, transcription corepressor activity and MHC protein complex binding. In addition, the main five enrichment pathways of GO cellular component (CC) were dense core granule, neuronal dense core vesicle, MHC class II protein complex, cytosol and spindle. The main five enrichment pathways for KEGG were autoimmune thyroid disease, staphylococcus aureus infection, EGFR tyrosine kinase inhibitor resistance, PPAR signaling pathway and prostate cancer. The main five enrichment pathways for reactome were translocation of ZAP-70 to Immunological synapse, PD-1 signaling, platelet Aggregation (Plug Formation), phosphorylation of CD3 and TCR zeta chains and regulation of complement cascade. The main five enrichment pathways for Wiki were 2q13 copy number variation syndrome, peptide GPCRs, pluripotent stem cell differentiation pathway, endoderm differentiation and overview of proinflammatory and profibrotic mediators.

**Figure 12:**
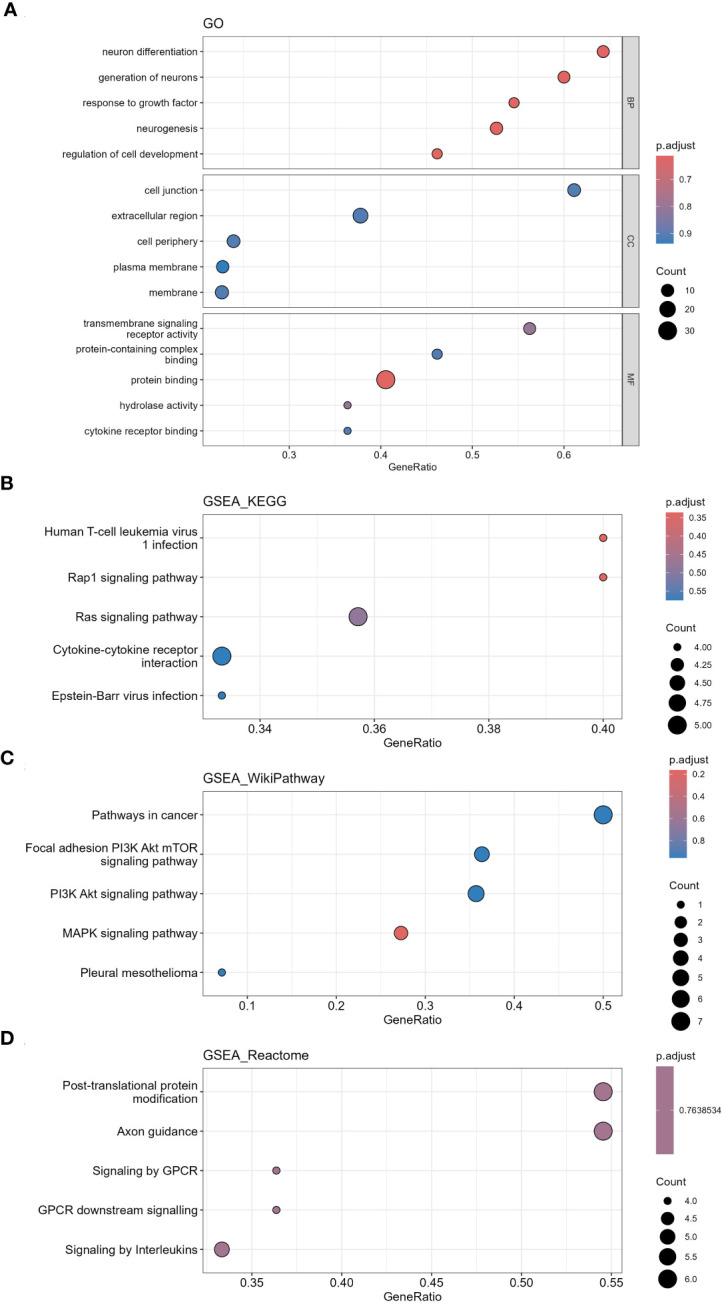
Functional annotation and pathway enrichment analysis of differentially expressed mRNAs in LUAD cohort via GSEA. **(A)** GO functional annotation, **(B)** KEGG pathway enrichment, **(C)** Wiki pathway enrichment, **(D)** Reactome pathway enrichment.

1212 mRNAs in LUAD cohort were analyzed for GO functional annotation and KEGG pathway enrichment. The results of the top 5 pathways for each enrichment analysis are shown in **Figure 12** and **Supplementary Table S9**. The main five enrichment pathways for GO biological processes (BP) were regulation of cell development, neurogenesis, generation of neurons, neuron differentiation and response to growth factor. The main five enrichment pathways of GO cellular component (CC) were cell junction, membrane, cell periphery, extracellular region and plasma membrane. The main five enrichment pathways of GO molecular function (MF) were transmembrane signaling receptor activity, protein binding, hydrolase activity, protein-containing complex binding and signaling receptor activity. The main five enrichment pathways for KEGG were human T-cell leukemia virus 1 infection, Rap1 signaling pathway, Ras signaling pathway, Epstein-Barr virus infection and Cytokine-cytokine receptor interaction. The main five enrichment pathways for reactome were signaling by GPCR, GPCR downstream signaling, post-translational protein modification, axon guidance and nervous system development. The main five enrichment pathways for Wiki were MAPK signaling pathway, PI3K Akt signaling pathway, focal adhesion PI3K Akt mTOR signaling pathway and pleural mesothelioma.

### 3.6 Analysis of tumor-infiltrating immune cells changes in different STK11 mutation status in lung cancer patients

**Figure 13:**
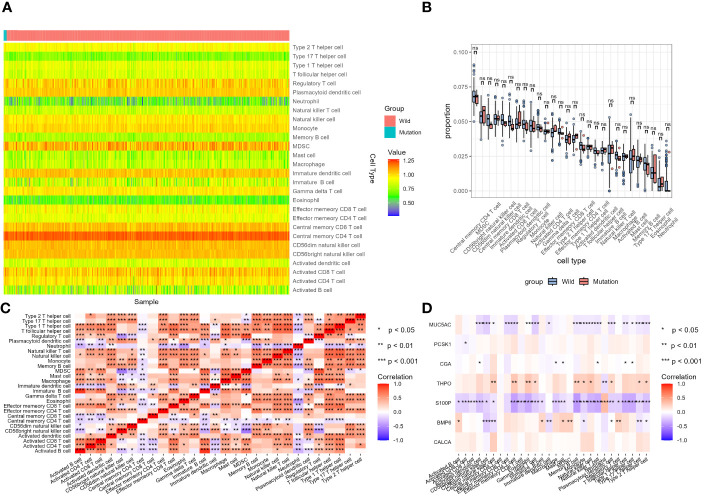
Analysis of tumor-infiltrating immune cells changes in different STK11 mutation status in LUSC cohort. **(A)** Heatmap of immune cell infiltration proportion of different samples, **(B)** Differential analysis of immune cell infiltration between wild-type and mutant STK11 samples, **(C)** Heatmap of correlation analysis of different immune cell infiltration, **(D)** Heatmap of correlation analysis between immune genes and immune cell infiltration.

**Figure 14:**
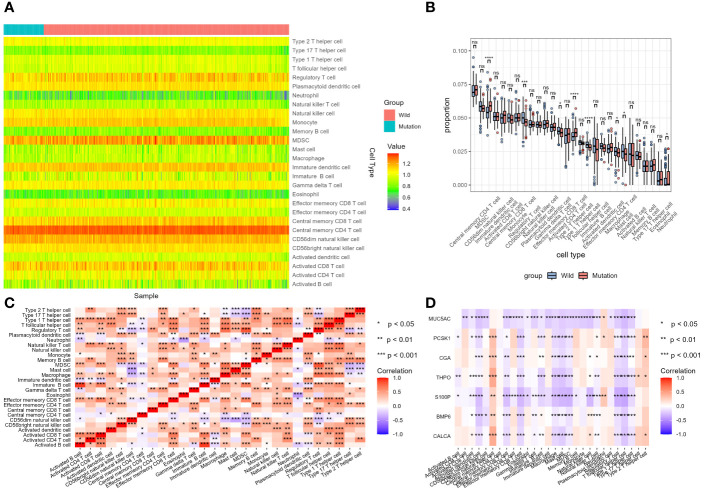
Analysis of tumor-infiltrating immune cells changes in different STK11 mutation status in LUAD cohort. **(A)** Heatmap of immune cell infiltration proportion of different samples, **(B)** Differential analysis of immune cell infiltration between wild-type and mutant STK11 samples, **(C)** Heatmap of correlation analysis of different immune cell infiltration, **(D)** Heatmap of correlation analysis between immune genes and immune cell infiltration.

ssGSEA results for LUSC cohort were shown in **Figures 13A–D**. Enrichment score results of immune gene set in TCGA samples were shown in **Supplementary Table S10**. None of the 28 immune cells showed statistical difference in LUSC cohort between STK11 wild-type LUSC patients and STK11 mutation-type LUSC patients, shown in **Supplementary Table S11**. However, apart from CALCA, the 6 IRGs we screened in 3.4 part were significantly correlated with the immune infiltration enrichment scores of 28 immune cells. ssGSEA results for LUAD cohort were shown in **Figures 14A–D**. Enrichment score results of immune gene set in TCGA samples were shown in **Supplementary Table S12**. Effector memory CD8 T cell, macrophage, mast cell, neutrophil, regulatory T cell, CD56 dim natural killer cell, Type 2 T helper cell and T follicular helper cell were showed statistical difference in LUSC cohort between STK11 wild-type LUSC patients and STK11 mutation-type LUSC patients, shown in **Supplementary Table S13**. And more, 7 IRGs screened in 3.4 part were significantly correlated with the immune infiltration enrichment scores of 28 immune cells.

## 4 Discussion

Our present study demonstrated that low expression of STK11 protein and presence of STK11 mutation was associated with poor prognosis in NSCLC, combining real-world cohort and publicly available databases to comprehensively evaluate the clinical impact of this gene on the prognosis of NSCLC patients based on protein expression and gene mutation data.

Clarifying the contribution of somatic gene mutation status and mechanism to cancer is crucial for personalized precision medicine, which has been confirmed by clinical experience in targeted therapy (19, 20). STK11 is a tumor suppressor and a negative regulatory factor as target of rapamycin signaling in mammalians (21). It’s inactivated in 30-35% of cancer cell lines, but only in 5-15% of primary cancer types (19). Nonetheless, the important biological role of STK11 had attracted widespread attention. It’s demonstrated that typical tumor suppressive effect of STK11 involved the activation of AMPK-related kinases reckoned as major regulators of cell survival under conditions of stress (22). In preclinical models, STK11 inactivation often led to cancer progression and metastasis and was associated with indolent tumor immune microenvironment, exhibiting as a reduced density of infiltrating cytotoxic CD8^+^ T lymphocytes, decreased PD-(L)1 expression and a neutrophil-rich tumor microenvironment (7). Loss of STK11 function was identified as a potential feature of malignant tumors in a variety of malignancies, such as cervical cancer (23), meningiomas (24), cholangiocarcinoma (25) and lung cancer (13). STK11 affects tumor cell growth through various important cellular pathways, and gene mutations can affect pathways such as AMPK, STING, and vascular endothelial growth factor, leading to immune suppression and changes in the metabolic environment, which resulted in tumor growth (10). A recent study suggested that STK11 mutation might affect the killing effect of NK cell and promoted progression and metastasis in LUAD (26). Based on the above situation, STK11 is an important protective factor for cancer patients, and its loss of function or genetic mutations may lead to poor prognosis for cancer patients. Therefore, exploring the mechanism of action of STK11 in cancer patients is of great clinical value for a deeper understanding of the biological role and prognosis of STK11.

In our current study, we had explored the prognostic value of STK11 in NSCLC through two aspects of research, and combined with bioinformatics analysis, predicted and analyzed the potential mechanism of STK11 mutations causing deterioration of cancer biological behavior. First of all, we integrated the immunohistochemical data of STK11 in NSCLC patients, and grouped them based on expression scores to compare their survival according to expression levels of STK11. Similar to previous research findings and consistent with our expectations, we found that high expression of STK11 was a protective factor for a good prognosis (27). Our result showed that the risk of death in patients with high expression of STK11 decreased by 58%, while the risk of cancer recurrence and metastasis decreased by 64%. The research results of our cohort further supported the anticancer effect of STK11. Further data analysis indicated that high expression of STK11 was an independent factor affecting overall survival and progression free survival in NSCLC patients. Previous research has showed that expression of STK11 was positively correlated with intertumoral infiltration of cluster of differentiation CD3^+^, CD4^+^, and CD8^+^ cells, demonstrating patients with high levels of STK11 would have better immune conditions for tumor control (27). It’s also found that patients with extra-thoracic recurrence had lower tumor expression of STK11 than those with intrathoracic recurrence. It can be found that in multivariate analysis, low STK11 expression remained independently associated with poor disease-free survival and distant disease-free survival (27).

Secondly, we obtained mutation data of the STK11 gene from patients in the LUSC cohort and LUAD cohort from TCGA. Further analysis showed that the mutation rate of STK11 in LUSC cohort was much lower than that in LUAD cohort. Only 1.04% (5/482) patients were found to have STK11 mutations in LUSC cohort and 16.70% (74/443) patients were found to have STK11 mutations in LUAD cohort. Therefore, we speculated that the mutation significance of STK11 might be more promising in LUAD patients. Because the level of STK11 mutation in LUSC cohort is very low, this limits the application value of STK11 mutation in LUSC cohort, which may be the same as other mutant proteins, such as EGFR, ALK, etc. Thus, it may be more realistic to develop targeted drugs and therapeutic regimens against STK11 mutations for LUAD. Some existing data showed that the proportion of STK11 mutations in NSCLC is about 5–30% (28), and the proportion of patients with advanced or metastatic NSCLC may be higher (29, 30), demonstrating that STK11 mutation might probably be a late event in the evolvement of carcinogenesis in lung cancer. Notably, STK11 mutations have been associated with poor outcomes in NSCLC patients treated with immune checkpoint inhibitors (ICIs) (7, 31). According to the available literature, STK11 mutation is associated with the immunosuppressive tumor microenvironment, which acts as a barrier against some anti PD-1/PD-L1 antibodies in NSCLC (28, 31). In our study, both of all patients in LUSC cohort and LUAD cohort were divided into STK11^mut^ group and STK11^wt^ group. We found that the presence of STK11 mutation was significantly associated with shortened OS both in LUSC cohort and LUAD cohort. Rosellini et al. had also found that STK11 mutation is associated with poor prognosis in NSCLC in terms of OS and first-line time to treatment failure (TTF) (14). Another study reported the same results, that is, OS was significantly shorter for patients with STK11 mutation (STK11^Mut^ 14.2 months vs. STK11^Wt^ 27.0 months) (28). Among NSCLC patients, the STK11 mutation was associated with a worse outcome for patients receiving systemic antitumor therapy, but not immune checkpoint inhibition therapy (13). Consistent with previous reports, our findings indicated that the presence of STK11 mutation was associated with poor prognosis in NSCLC. It should be noted that a large proportion of LUAD patients have multi-gene co-mutations, presenting with significantly different prognoses (32). For instance, STK11 may have co-mutations with KRAS, KEAP1 or EGFR (8, 13, 29, 32, 33), and some real-world study showed that patients with co-mutation of STK11 and KEAP1 or KRAS was associated with significantly shorter survival (33).

The evaluation of tumor immune microenvironment (TIME) has become an important target for tumor prevention and treatment, as there is a large amount of data supporting the prognosis and potential predictive significance of tumor infiltrating lymphocytes in various types of tumors (34). So far, tumor infiltrating lymphocytes (TILs) have been shown to be an indicator of ICIs treatment in PD-L1 positive patients (20). The strongest prognostic marker for tumor infiltrating lymphocytes in NSCLC is CD8 (+) T lymphocytes (35). Cytotoxic CD8 (+) T lymphocytes can directly kill cancer cells, while CD4 (+) T lymphocytes are involved in the activation and inhibition of CD8 (+) T lymphocytes (36). The STK11 gene, as one of the most important tumor suppressors in NSCLC, its functional deficiency may be a key factor in regulating the tumor immune microenvironment (37). Mutations of STK11 gene in NSCLC are associated with poor patient responses to ICIs, and mechanistically, this occurred because STK11 mutant NSCLCs lacked TCF1-expressing CD8 T cells, a phenotype recapitulated in human STK11 mutant NSCLCs. One study has shown that systemic inhibition of type I interferon secretion by Axl can lead to the expansion of tumor associated TCF1+PD-1+CD8 T cells and restore the therapeutic response of lung cancer to ICIs (38). The immune microenvironment of STK11 mutant tumors was mainly characterized by high neutrophil density, while the density of CD8 (+) T lymphocytes in the stroma was low (39). Hiraoka et al. demonstrated that high levels of CD8 (+) T lymphocytes and CD4 (+) T lymphocytes were important indicators for evaluating the prognosis of NSCLC patients, and the cooperation between these cell populations may produce more effective anti-tumor responses than any individual population (40). Wang et al. found that through the TIMER and TISIDB databases that infiltrating immune cells, including B cells, CD8 (+) T lymphocytes, CD4 (+) T lymphocytes, macrophages, and dendritic cells, were significantly reduced in patients with STK11 mutations, which indicate that patients carrying STK11 mutations might have a cold tumor immune microenvironment or immune desert type, and therefore could not benefit from immunotherapy (41, 42). As a whole, it is not clear how STK11 mutations affect the tumor immune microenvironment. Previous study has shown that STK11 mainly encodes serine threonine kinase, which regulates cell metabolism, energy homeostasis, cell growth, etc. through AMPK signaling pathway and 12 AMPK related kinases (12). In this study, we extracted gene expression profiles of STK11^Mut^ and STK11^WT^ patients from the LUSC cohort and LUAD cohort from TCGA, and focused on IRGs. We had identified a total of 7 IRGs with differential expression between STK11^Mut^ and STK11^WT^ patients. Our results suggested that IRGs might be one of the reasons why STK11 causes changes in the tumor microenvironment. It is worth noting that in our study we found that STK11 mutations were not significantly associated with tumor infiltrating cells in the LUSC cohort, but in LUAD cohort. The difference in immune infiltrating cells may be an important reason for the different prognosis of STK11 mutations and STK11 wild-type.

Our finding strengthened the idea that low expression of STK11 protein and presence of STK11 mutation was associated with poor prognosis in NSCLC. And from the perspective of bioinformatics analysis, the differential expression of IRGs in the population with STK11 mutations was preliminarily revealed. Some limitation of our study should be mentioned. First of all, this is an observational study. Although all patient cases make relevant treatment decisions based on international recommendations, there is heterogeneity in the exact regimen of radiotherapy or chemotherapy for patients, which may affect their progression free survival and overall survival. Second, the low sample size may be a potential limitation of this study. In the future, prospective studies with large samples may need to be further carried out to verify our results. Third, the paraffin tissue related to STK11 detection was selected in early-stage and locally advanced lung cancer, the reason for which is that many metastatic lung cancers do not have the opportunity of surgery, and similar paraffin tissue cannot be obtained. Therefore, further prospective studies are needed to verify the prognostic value of STK11 expression in metastatic lung cancer in the future. Fourth, we have preliminarily explored the possible mechanisms of IRGs in STK11 mutations through gene difference analysis and gene function enrichment analysis, but further research is still needed to reveal the mechanisms of tumor microenvironment changes caused by STK11 mutations. Similarly, prospective experiments need to be carried out to verify the effect of STK11 gene mutation on the expression profile of IRGs. As insights on future directions, the expression profile differences of different STK11 gene mutation status can be verified by whole transcriptome sequencing technology or whole exome sequencing technology. In addition, reverse transcription-polymerase chain reaction (RT-PCR) and Western Blotting experiments can be further applied to explore the differential expression of key IRGs at the RNA and protein levels.

## 5 Conclusion

In conclusion, low expression of STK11 protein and presence of STK11 mutation is associated with poor prognosis in NSCLC. The STK11 mutation may alter the expression profile of IRGs, which may explain the changes in the immune microenvironment of STK11Mut, which deserves further investigation.

## Data availability statement

The original contributions presented in the study are included in the article/**Supplementary Material**. Further inquiries can be directed to the corresponding author.

## Ethics statement

The studies involving humans were approved by the ethics committee of Fujian Medical University Cancer Hospital. The studies were conducted in accordance with the local legislation and institutional requirements. The participants provided their written informed consent to participate in this study.

## Author contributions

JZ: Writing – review & editing, Writing – original draft, Validation, Funding acquisition, Data curation. YD: Writing – original draft, Formal Analysis, Data curation. BH: Writing – review & editing, Investigation, Formal Analysis, Data curation. XC: Writing – original draft, Validation, Funding acquisition, Formal Analysis.

## Supplementary material

Supplementary Figure 1 Random forest decision trees for survival analysis.

Supplementary Figure 2 The requested performance error and variable importance for random forest model.

Supplementary Figure 3 Estimates of survival for random forest model. **(A)** Survival estimates for each individual (only first 5 individuals were shown), **(B)** Brier score, **(C)** Continuous rank probability score (CRPS) =brier score/time, **(D)** Relationship between individual mortality and observation time.

Supplementary Figure 4 Curve of Brier score over time.

Supplementary Figure 5 The variable importance for random forest model.
