# Supplementary figures and images for: Prognostic implications of STK11 with different mutation status and its relationship with tumor-infiltrating immune cells in non-small cell lung cancer
Source: Front Immunol. 2024 Apr 26;15:1387896. doi: 10.3389/fimmu.2024.1387896 (PMC11082287; doi:10.3389/fimmu.2024.1387896)

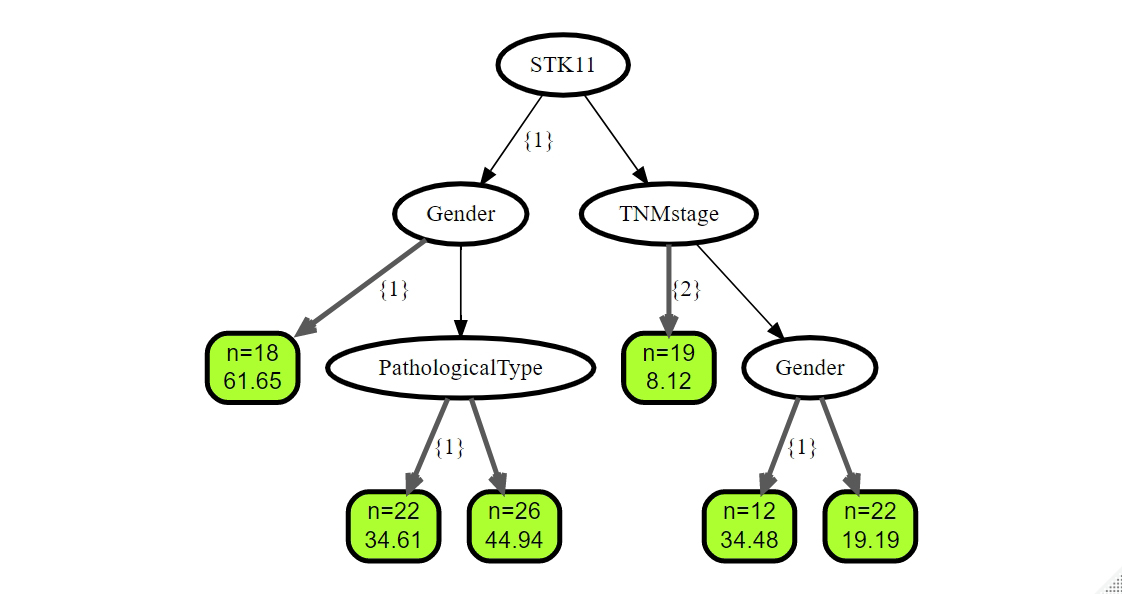

Supplement: Supplementary Figure 1 — Random forest decision trees for survival analysis. [file Image_1.jpeg]

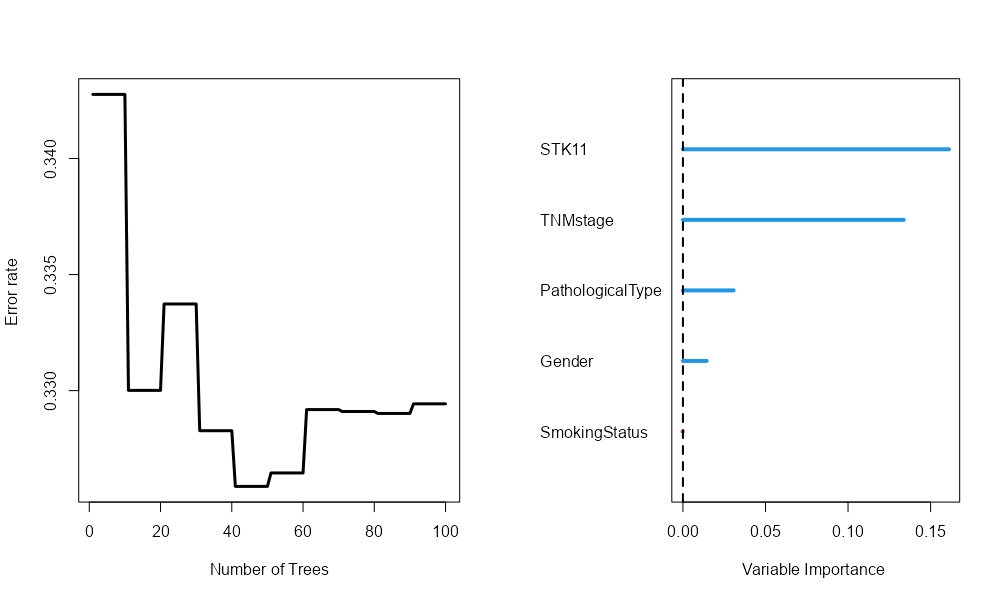

Supplement: Supplementary Figure 2 — The requested performance error and variable importance for random forest model. [file Image_2.jpeg]

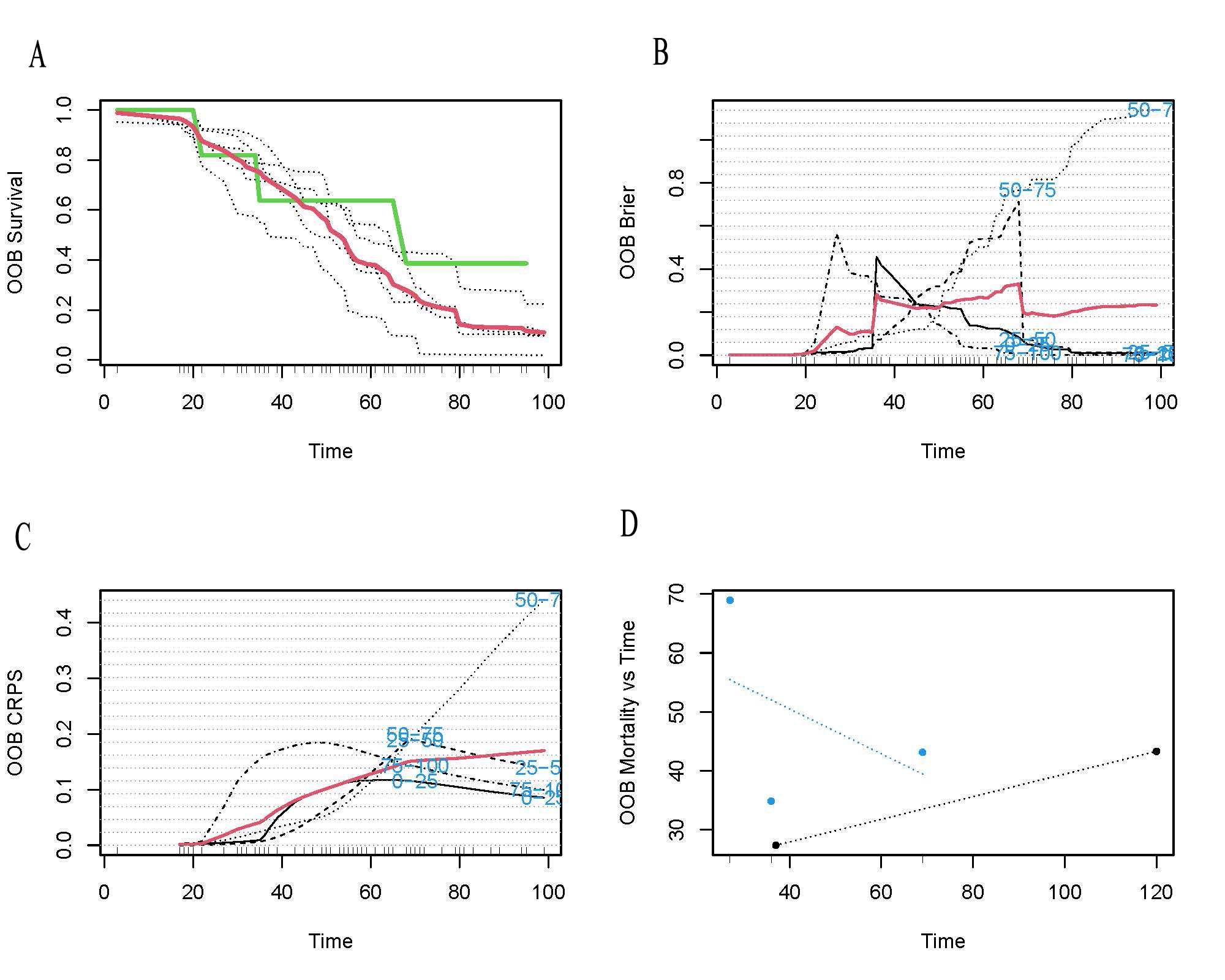

Supplement: Supplementary Figure 3 — Estimates of survival for random forest model. (A) Survival estimates for each individual (only first 5 individuals were shown), (B) Brier score, (C) Continuous rank probability score (CRPS) =brier score/time, (D) Relationship between individual mortality and observation time. [file Image_3.jpeg]

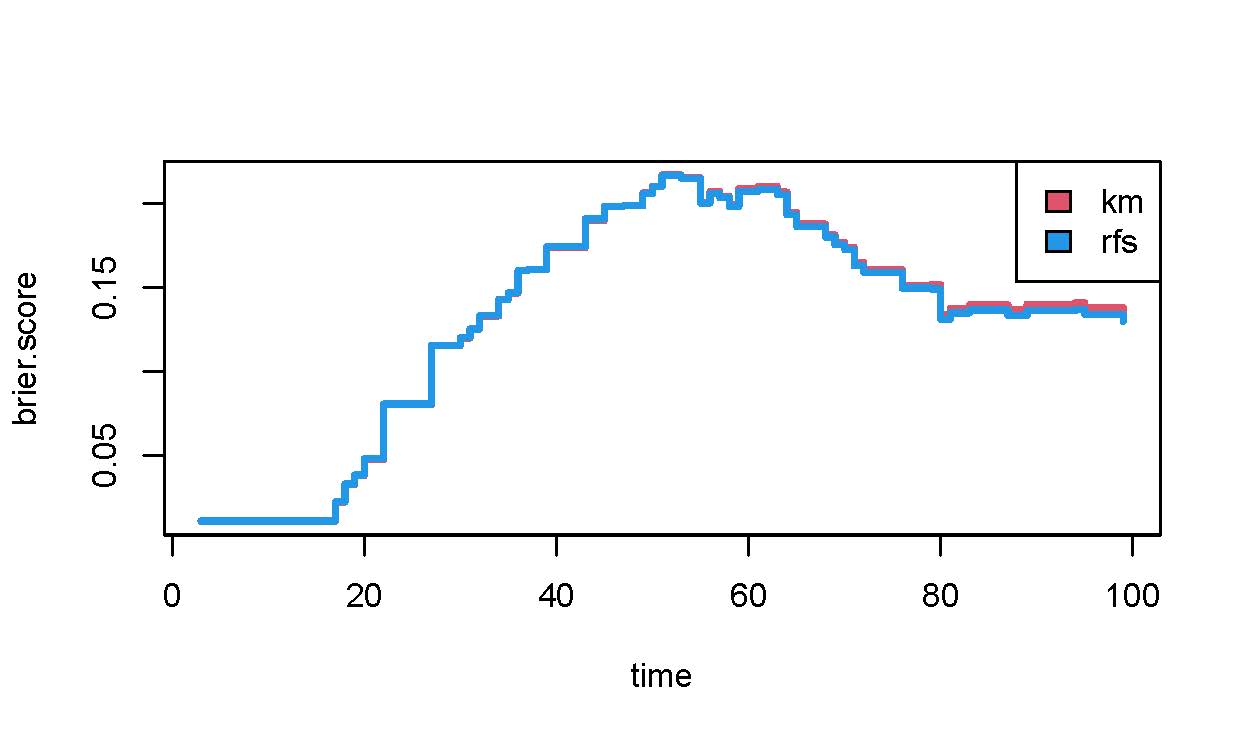

Supplement: Supplementary Figure 4 — Curve of Brier score over time. [file Image_4.jpeg]

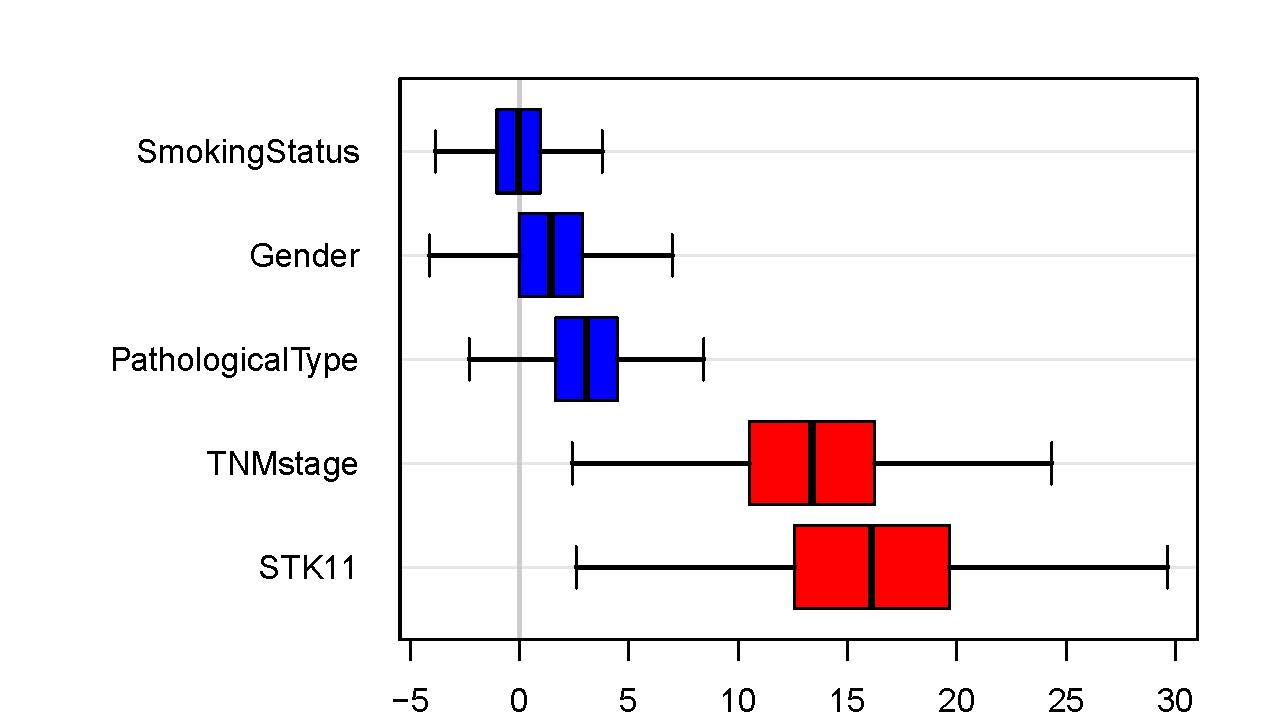

Supplement: Supplementary Figure 5 — The variable importance for random forest model. [file Image_5.jpeg]
